# Spontaneous intracranial artery dissection: risk factors, clinical features and imaging features

**DOI:** 10.1080/07853890.2026.2634573

**Published:** 2026-02-24

**Authors:** Yidi Wang, Qingqing Jiang, Xiang Chen, Qiming Liang, Shiyi Cao, Furong Wang

**Affiliations:** a Department of Neurology, Tongji Hospital, Tongji Medical College, Huazhong University of Science and Technology, Wuhan, Hubei, China; b School of Nursing, Tongji Medical College, Huazhong University of Science and Technology, Wuhan, Hubei, China; c School of Public Health, Tongji Medical College, Huazhong University of Science and Technology, Wuhan, Hubei, China

**Keywords:** Spontaneous intracranial artery dissection, risk factors, clinical features, imaging features, case-control study

## Abstract

**Purpose:**

There remains a lack of epidemiological data and evidence regarding risk factors for intracranial arterial dissection (IAD) worldwide, making it difficult to make a more timely and accurate clinical diagnosis. We aimed to identify risk factors, clinical and imaging features of spontaneous IAD (sIAD) using a case-control design.

**Methods:**

We collected data on sIAD patients admitted to Tongji Hospital in Wuhan, China from June 2017 to June 2024. Non-IAD ischemic stroke (IS) and non-IAD intracerebral hemorrhage (ICH) patients served as control groups. Logistic regression models analyzed the three data sets, with results expressed as odds ratio (OR) and 95% confidence interval (CI).

**Results:**

After screening, 71 patients with sIAD, 84 patients with non-IAD IS and 102 patients with non-IAD ICH were included in this study. The findings showed that the participants with diabetes had a lower likelihood of sIAD than non-IAD IS (OR = 0.145, 95%CI = 0.030–0.702). Compared with non-IAD ICH patients, individuals with sIAD had lower systolic blood pressure on admission (OR = 0.941, 95%CI = 0.900–0.983) and less likely to be young (OR = 0.911, 95%CI = 0.855–0.970). Serological data showed that compared with non-IAD ICH patients, elevated triglyceride (OR = 0.326, 95%CI = 0.179–0.594) were associated with the reduced likelihood of sIAD, whereas the opposite was true for uric acid levels (OR = 1.007, 95%CI = 1.000–1.014). In imaging, sIAD patients showed the largest number of arterial lumen dilatation, followed by stenosis with dilatation.

**Conclusions:**

Diabetes may be associated with a reduced likelihood of sIAD. Differences in serologic markers may help in the differential diagnosis of sIAD from other cerebrovascular events.

## 1. Introduction

Cerebral artery dissection (CAD) is one of the main causes of ischemic stroke (IS) in young and middle-aged people, accounting for 10%-25% [1,2]. According to the site of occurrence, CAD can be categorized into intracranial artery dissection (IAD) and extracranial artery dissection (EAD) [3]. The incidence of EAD is 3.6 to 4.4 cases per 100,000 people per year [3,4], while the incidence of IAD is still unclear [5]. Studies have shown that the incidence of IAD varies by geographical location. For example, the proportion of IAD in CAD is about 11% in the European population [6], while it can be as high as 67-78% in the East Asian population [7]. At present, although imaging findings are recommended as one of the diagnostic criteria of IAD in the world [8,9], a unified and clear diagnostic method has not been established. Research on relevant risk factors and diagnostic approaches remains limited. Furthermore, existing studies are predominantly small-sample retrospective analyses [10,11], which often pool spontaneous and traumatic cases, leading to high population heterogeneity. Additionally, these studies mostly focus on single diseases or specific subtypes, lacking systematic integration of clinical characteristics and risk factor analyses, and thus have limitations in constructing differential diagnosis models and analyzing risk factors. Further research is needed to enhance the clinical understanding and evidence improvement of IAD. If IAD is not treated in time, it may continue to develop into cerebral infarction and subarachnoid hemorrhage with high disability and mortality [12–14]. Therefore, timely and accurate diagnosis and discovery of IAD is of great help to improve the prognosis of patients. Building upon previous retrospective studies, this study selected patients with spontaneous IAD (sIAD: spontaneous means that the patient ‘s dissection is not caused by direct or indirect mechanical impact on the neck area) as subjects to minimize the influence of external factors on the results. In addition, we used patients with non-IAD ischemic stroke (IS) and non-IAD intracerebral hemorrhage (ICH) as controls. Based on the analysis of the clinical and imaging characteristics of sIAD, we conducted an in-depth comparison of cerebrovascular risk factors among these three conditions, whose incidence rates are closely linked to cerebral vascular health [15–17]. This is complementary to previous studies that only focused on the characteristics of IAD single disease or limited to its specific classification, aiming to provide a more targeted clinical basis for the early diagnosis and prevention of sIAD.

## 2. Subjects and methods

**Figure 1.**
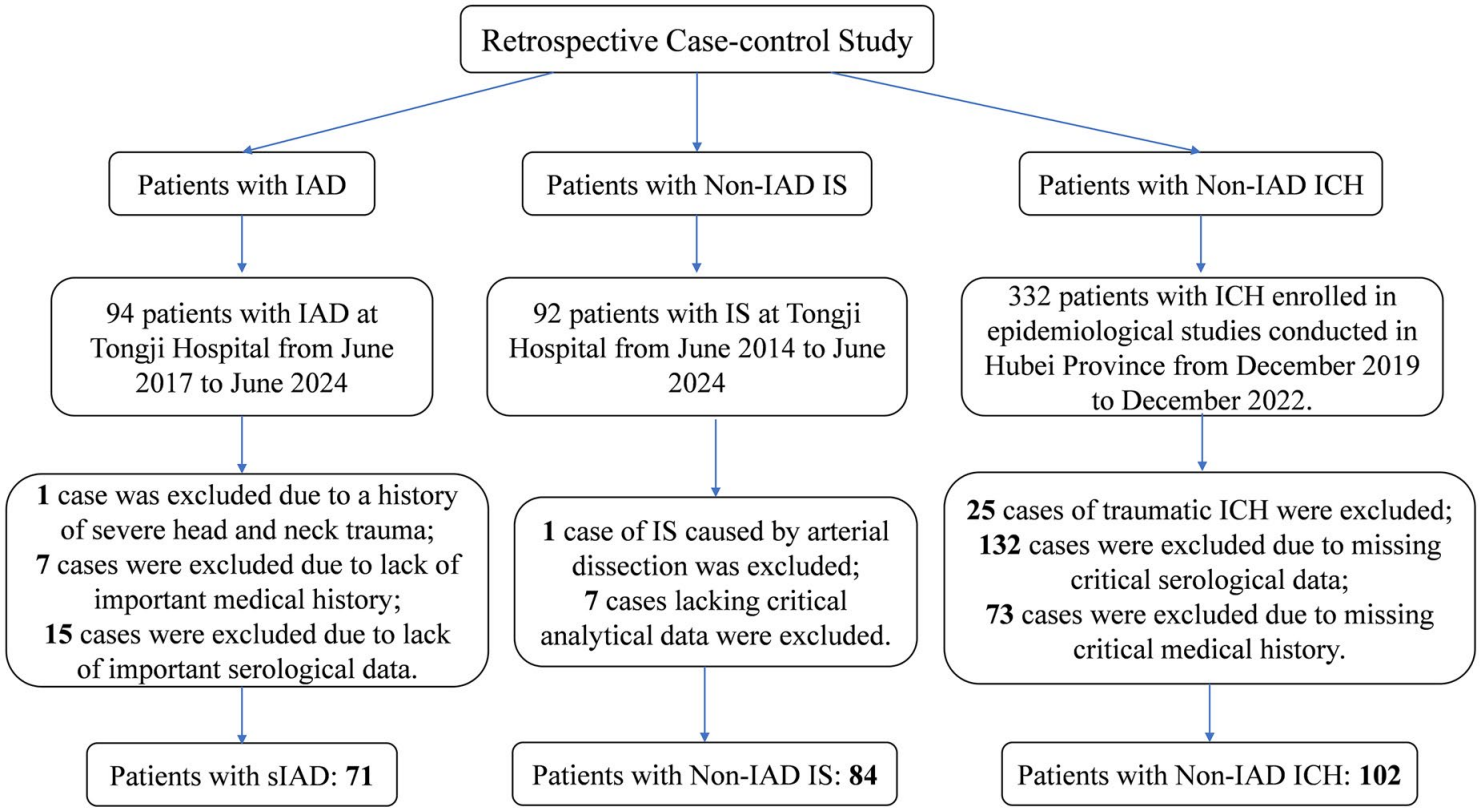
Flowchart of case selection for the retrospective case-control study. 1. sIAD patient group: 94 IAD patients were initially included in Tongji Hospital from June 2017 to June 2024, of which 1 was excluded due to a history of severe head and neck trauma, 7 were excluded due to lack of important medical history, and 8 were excluded due to lack of important epidemiological data. Finally, 71 sIAD patients were included. 2. Non-IAD IS group: 92 patients with IS were initially included in Tongji Hospital from June 2014 to June 2024, of which 1 patient was excluded due to IS caused by arterial dissection, and 6 patients were excluded due to missing analysis data. Finally, 85 patients with non-IAD IS were included. 3. Non-IAD ICH group: 332 patients with ICH were initially included in the epidemiological study of Hubei Province from December 2019 to December 2022, of which 25 were excluded due to traumatic cerebral hemorrhage, 132 were excluded due to the lack of key clinical data, and 73 were excluded due to the lack of key medical history. Finally, 102 patients with non-IAD ICH were included.

This study conducted a retrospective case-control study involving the following three groups of individuals: (1) patients with sIAD, (2) patients with acute IS caused by causes other than IAD (Non-IAD IS), and (3) patients with ICH caused by causes other than IAD (Non-IAD ICH). The selection and exclusion of subjects in each group are shown in Figure 1. The study was approved by Research Ethics Committee of Tongji Hospital, and each participant signed informed consent forms (TJ-IRB202407041).

### 2.1. Patients with sIAD

A total of 94 patients who were diagnosed with IAD at Tongji Hospital Affiliated to Huazhong University of Science and Technology, Hubei Province, China, from June 2017 to June 2024 were retrospectively included in this study, and their medical history data and laboratory data were collected. Among the 94 patients with IAD included in this study, one case was excluded due to a history of severe head and neck trauma within the preceding 4 weeks (e.g. resulting in skull or cervical spine fractures, hemorrhage, etc.), thereby ruling out exogenous pathogenic factors. Seven patients were excluded due to lack of important medical history, and 15 patients were excluded due to lack of important serological data. Finally, we included 71 patients with sIAD. All enrolled patients met the following inclusion criteria: (1) patients with confirmed diagnosis of sIAD in accordance with the consensus of sIAD diagnosis by combining the patient’s medical history, clinical symptoms, signs and imaging manifestation [7]; (2) complete medical history data and laboratory data; (3) age ≥ 18 years. Exclusion criteria:(1) patients with definite severe head and neck trauma resulting in arterial dissection; (2) the clinical data required for the study were incomplete; (3) other diseases with clinical and imaging manifestations similar to those of IAD, including 1) atherosclerotic stenosis and thrombosis after rupture of atherosclerotic plaques; 2) vasospasm due to subarachnoid hemorrhage; 3) pseudoaneurysms; 4) vasculitis; 5) fusiform aneurysms without dissection, etc [7].

### 2.2. Patients with non-IAD IS

This group was patients with acute IS caused by something other than IAD. We retrospectively included a total of 92 patients diagnosed with IS at Tongji Hospital from June 2014 to June 2024. After screening, one case of IS caused by arterial dissection was excluded, along with 7 cases lacking critical analytical data. Ultimately, medical histories and laboratory data were collected from 84 patients. All enrolled patients met the following inclusion criteria: (1) all acute IS diagnoses met the consensus criteria for IS diagnosis [18], with imaging studies ruling out sIAD; (2) availability of complete medical history and laboratory test data; (3) age ≥ 18 years. Exclusion criteria: (1) imaging studies indicating IAD, or clinical determination that the direct cause of IS was lumen stenosis or occlusion due to sIAD; (2) incomplete clinical data required for this study; (3) patients with a prior diagnosis of sIAD.

### 2.3. Patients with non-IAD ICH

Subjects in this group were patients with ICH caused by non-IAD causes. We included data from the epidemiological survey records of patients with ICH conducted in Hubei Province, China from December 2019 to December 2022. A total of 332 patients with ICH were included, and no dissection was found in the imaging data of all patients. Among these, 25 cases of traumatic ICH were excluded, 132 cases were excluded due to missing critical serological data, and 73 cases were excluded due to missing critical medical history. A total of 102 cases were included in this group. All enrolled patients met the diagnostic and treatment guidelines for ICH and were radiologically excluded from sIAD; data were complete; age ≥ 18 years. Exclusion criteria were as follows: (1) radiological examination showing typical features of IAD, or clinical judgment indicating ICH related to sIAD (e.g. subarachnoid hemorrhage due to dissecting aneurysm rupture followed by intracerebral hematoma); (2) incomplete clinical data; (3) secondary ICH caused by identifiable etiologies such as traumatic brain injury, brain tumors, or vascular malformations.

### 2.4. Collection of patient data

**Figure 2.**
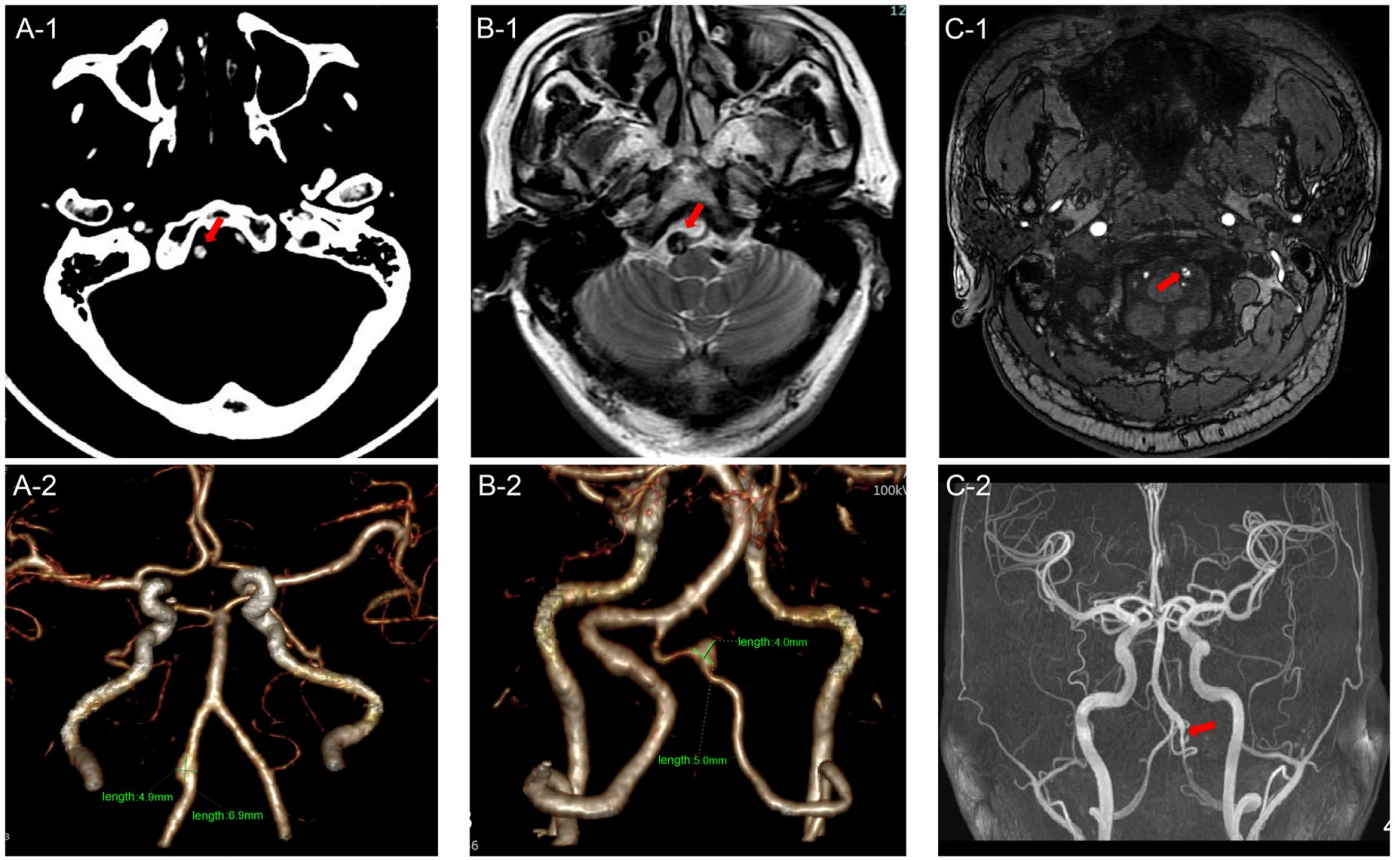
The imaging features of spontaneous intracranial artery dissection. A. CTA angiography of the head and neck in a 62-year-old male patient. (A-1) CTA raw thin-layer image showing a linear irregular isodense intima-media flap shadow visible in the lumen of the right vertebral artery (arrowed). (A-2) CTA volume reconstruction showed local tumor-like expansion of the right vertebral artery, with a size of about 7*5mm. B. A 54-year-old male patient with a diagnosis of limited dissection of the V4 segment of the right vertebral artery. (B-1) Magnetic resonance diffusion weighted imaging of the head showed high signal intima flap of the right vertebral artery (arrowed). (B-2) CTA volume reconstruction showed local nodular protrusions in the right vertebral artery V4 segment, with a size of about 4*5mm. C. High-resolution magnetic resonance enhancement imaging of the cervical and intracranial arterial vessel walls in a 49-year-old male patient. (C-1) Low and short T1 signal double-lumen shadows were seen in the proximal segment of the left vertebral artery, and the local true lumen was severely narrowed(arrowed). (C-2) 3D time-of-flight MRA (TOF⁃MRA) shows stenosis with dilatation of the intracranial segment of the left vertebral artery(arrowed).

We collected the demographic characteristics (age, sex), potential risk factors (body mass index (BMI), hypertension, diabetes, coronary heart disease, admission season (divided according to the month of hospitalization: March to May as spring, June to August as summer, September to November as fall, and December to February as winter)) [19], and clinical features (neurological related symptoms (headache, vertigo, limb hypoesthesia, limb weakness, dyskinesia, limb seizures, dysarthria, nausea, vomiting), heart rate, respiratory rate, blood pressure, whether or not surgery was performed, whether or not medications were used, whether or not anticoagulant and antiplatelet medications were used, and the indicators of the first blood tests after admission to the hospital (routine blood tests, liver function tests, renal function tests, blood lipids, blood glucose, electrolytes, and coagulation function tests)) of patients in each group. We also collected information on the patient’s imaging presentation (computed tomography angiography (CTA), magnetic resonance angiography (MRA)) as shown in Figure 2.

### 2.5. Statistical analyses

Quantitative data with normal distribution (including approximate normal distribution) were expressed as mean ± standard deviation ($\bar{x}\pm s$), and the analysis of variance (ANOVA) was used for comparison between groups. The categorical data were expressed by percentage and frequency, and the comparison between groups was performed by χ^2^ test. Subsequently, we included the variables with *p* < 0.10 in the above difference analysis into the multivariate logistic regression (generalized logit) model to compare the influencing factors of the three groups (patients with sIAD, patients with non-IAD IS, patients with non-IAD ICH). The odds ratio (OR) was calculated by the constructed logistic regression model, and the variables were gradually selected to determine the independent predictors related to the incidence of sIAD. Based on the results of the above data, we also conducted subgroup analyses of age (whether or not greater than 60 years old), sex, and BMI values(less than 18.5 kg/m^2^ was considered underweight, 18.5 kg/m^2^-24 kg/m^2^ was considered normal, 24 kg/m^2^-28kg/m^2^ was considered overweight, and more than 28 kg/m^2^ was considered obese) between the sIAD group and the non-IAD IS group using diabetes as an exposure factor, which we also used the logistics regression model for analysis, and used R software version 4.2.1 to draw a forest map for subgroup analysis. In addition, we also divided the sIAD group into two specific subgroups according to the location of the dissection: intracranial internal carotid artery dissection and intracranial vertebral artery dissection. Referring to the same method above, we first analyzed the differences of each variable, and selected the appropriate variables into the binary logistic regression model to evaluate the heterogeneity of the odds ratio between the two subgroups. The results were expressed as OR and its 95% confidence interval (CI). Statistically significant differences were defined as *p* ≤ 0.05 for the two-sided test. We used SPSS version 27.0 for statistical analysis of the data.

## 3. Results

The average age of the sIAD group was 55.49 ± 2.78 years, and 46 (64.8%) were male. Based on the imaging findings, all 71 patients belonged to sIAD, with a slight predominance in the number of patients with intracranial internal carotid artery dissection (37, 52.11%). Among the imaging modalities employed, combined CTA and DSA was the most prevalent approach (26, 36.6%). For single imaging modalities, DSA served as the primary diagnostic method (9, 12.7%), followed by CTA (5, 7.0%). A total of 50 patients underwent CTA, 55 patients underwent DSA and 27 patients underwent MRA. Of these, the largest number of CTA imaging findings showed luminal dilatation (tumor-like changes) (35, 70%), and this occurred equally in DSA (38, 69.1%) and MRA (11, 40.7%). There were 9 patients (18%) whose CTA imaging results showed no dissection, and 4 patients (14.8%) in MRA, all of which needed to be further diagnosed by DSA. Among the imaging manifestations in the 55 patients with DSA, second only to the manifestation of luminal dilatation was the manifestation of stenosis with dilatation (10, 18.2%), i.e. bead-like changes.

We also included 84 patients with non-IAD IS and 102 patients with non-IAD ICH. The demographic characteristics, clinical characteristics and serological data of the three groups of subjects are shown in Table 1 and Table 2 (complete data is set out in Supplementary Table 1 and Supplementary Table 2). We included the variables with *p* > 0.1 into the multivariate logistic regression analysis model of the three groups (Table 3).

**Table 1.** Demographic characteristics and clinical features profile of the sIAD group, the non-IAD IS group, and the non-IAD ICH group.

Variable	sIAD (*N* = 71)	IS (*N* = 84)	ICH (*N* = 102)	*P*
Age (yr)	55.49 ± 2.78	55.02 ± 2.53	61.23 ± 2.65	0.001
Males	46(64.8%)	55(65.5%)	71(69.6%)	0.757
BMI (kg/m^2^)	24.57 ± 0.75	24.14 ± 0.65	24.15 ± 0.75	0.563
Admission Season				0.354
Spring	26(36.6%)	23(27.4%)	30(29.4%)	
Summer	16(22.5%)	20(23.8%)	25(24.5%)	
Autumn	17(23.9%)	17(20.2%)	30(29.4%)	
Winter	12(16.9%)	24(28.6%)	17(16.7%)	
Clinical Symptoms				
Vertigo	34(47.9%)	29(34.5%)	/	0.091
Limb Weakness	12(16.9%)	29(34.5%)	/	0.013
Dyskinesia	4(5.6%)	16(19%)	/	0.013
Slurred Speech	5(7.0%)	27(32.1%)	/	<0.001
Vomiting	7(9.9%)	17(20.2%)	/	0.075
Heart Rate(bpm)	77.28 ± 2.77	82.51 ± 2.79	82.07 ± 3.49	0.018
Systolic Pressure(mmHg)	130.21 ± 4.12	135.14 ± 4.62	151.59 ± 5.57	<0.001
Diastolic Pressure(mmHg)	82.18 ± 3.17	82.94 ± 2.96	89.22 ± 4.15	0.020
Hypertension	38(53.5%)	46(54.8%)	64(62.7%)	0.393
Diabetes	6(8.5%)	23(27.4%)	14(13.7%)	0.004
Coronary Heart Disease	6(8.5%)	6(7.1%)	4(3.9%)	0.423
Operative Treatment	54(76.1%)	27(32.1%)	29(28.4%)	<0.001
Medication	15(21.1%)	50(59.5%)	/	<0.001
Use of anticoagulant or antiplatelet drugs	53(74.6%)	31(36.9%)	/	<0.001

Abbreviations: sIAD = spontaneous intracranial artery dissection; IS = ischemic stroke; ICH = intracerebral hemorrhage (please refer to Supplementary Table 1 for complete data due to space limitations).

**Table 2.** Serological parameters in the sIAD group, the non-IAD IS group, and the non-IAD ICH group.

Serology variables	sIAD (*N* = 71)	IS (*N* = 84)	ICH (*N* = 102)	*P*
WBC (10^9^/L)	5.66 ± 0.33	9.60 ± 1.03	9.68 ± 0.74	<0.001
RBC (10^12^/L)	4.36 ± 0.12	4.29 ± 0.15	4.89 ± 1.02	0.400
HGB (g/L)	132.73 ± 3.69	131.53 ± 4.35	143.44 ± 21.52	0.449
PLT (10^9^/L)	208.49 ± 15.04	210.76 ± 14.97	201.49 ± 16.74	0.676
NEUT (10^9^/L)	3.48 ± 0.28	7.37 ± 1.04	7.73 ± 0.71	<0.001
RDW (%)	13.34 ± 0.22	13.76 ± 0.25	/	0.014
PT (s)	11.57 ± 0.20	12.08 ± 0.38	13.27 ± 0.32	<0.001
TT (s)	14.47 ± 0.38	14.23 ± 0.36	12.77 ± 1.45	0.074
FIB (mg/dL)	287.87 ± 16.67	343.04 ± 25.77	387.42 ± 29.12	<0.001
DD (ng/ml)	367.54 ± 131.77	668.63 ± 206.77	46.43 ± 18.56	<0.001
AST/ALT	1.14 ± 0.13	1.31 ± 0.14	1.37 ± 0.12	0.053
SOD (kU/L)	175.94 ± 5.54	160.57 ± 7.70	/	0.002
GLU (mmol/L)	5.48 ± 0.43	7.17 ± 0.63	7.06 ± 0.50	<0.001
BUN (mmol/L)	5.45 ± 0.37	5.99 ± 0.84	7.49 ± 1.41	0.016
CREA (μmol/L)	69.32 ± 3.89	79.80 ± 13.34	92.68 ± 22.11	0.160
UA (μmol/L)	350.88 ± 19.33	304.51 ± 25.28	295.86 ± 29.54	0.002
CO_2_ (mmol/L)	26.47 ± 0.83	24.72 ± 0.97	/	0.008
SA (mg/L)	576.99 ± 23.91	638.37 ± 33.87	/	0.004
CHOL (mmol/L)	3.80 ± 0.31	4.16 ± 0.30	3.71 ± 0.38	0.112
TG (mmol/L)	1.34 ± 0.16	1.85 ± 0.37	2.37 ± 0.35	<0.001
HDL (mmol/L)	1.08 ± 0.05	1.09 ± 0.08	1.11 ± 0.07	0.795
LDL (mmol/L)	2.16 ± 0.23	2.39 ± 0.26	2.61 ± 0.17	0.009
Na (mmol/L)	140.74 ± 0.57	139.33 ± 0.96	/	0.013

Abbreviations: sIAD = spontaneous intracranial artery dissection; IS = ischemic stroke; ICH = intracerebral hemorrhage; WBC = white blood cell count; RBC = red blood cell; HGB = hemoglobin; PLT = platelet count; NEUT = neutrophil count; RDW = red blood cell volume distribution width; PT = prothrombin time; TT = thrombin time; FIB = fibrinogen; DD = D-dimer; ALT = alanine aminotransferase; AST = aspartate aminotransferase; SOD = superoxide dismutase; GLU = glucose; BUN = blood urea nitrogen; CREA = creatinine; UA = uric acid; CO_2_=carbon dioxide; SA = sialic acid; CHOL = cholesterol; TG = triglyceride; HDL = high-density lipoprotein; LDL = low-density lipoprotein; Na = sodium(please refer to Supplementary Table 1 for complete data due to space limitations).

**Table 3.** Multiple logistic regression model for the sIAD, non-IAD IS, and non-IAD ICH groups.

Variable	sIAD vs IS OR (95%CI)	*P*	sIAD vs ICH OR (95%CI)	*P*	IS vs ICH OR (95%CI)	*P*
Age(yr)	0.995(0.940–1.052)	0.857	0.911(0.855–0.970)	0.004	0.921(0.868–0.976)	0.006
SBP (mmHg)	0.978 (0.933–1.026)	0.368	0.941(0.900–0.983)	0.007	0.962(0.930–0.995)	0.023
DBP (mmHg)	1.044(0.974–1.119)	0.221	1.014(0.952–1.080)	0.666	0.982(0.941–1.026)	0.428
Diabetes	0.145(0.030–0.702)	0.016	3.526(0.420–29.642)	0.246	0.600(0.090–4.007)	0.598
WBC (10^9^/L)	0.592(0.290–1.207)	0.149	0.800(0.317–2.017)	0.636	1.491(0.665–3.344)	0.332
NEUT (10^9^/L)	0.820(0.376–1.791)	0.619	0.524(0.203–1.349)	0.180	0.514(0.223–1.187)	0.119
PT (s)	0.938(0.618–1.425)	0.766	0.197(0.103–0.378)	<0.001	0.239(0.129–0.444)	<0.001
TT (s)	1.107(0.753–1.627)	0.605	1.180(0.996–1.398)	0.056	1.246(1.086–1.429)	0.002
AST/ALT	0.440(0.152–1.269)	0.129	0.484(0.145–1.619)	0.239	1.109(0.399–3.079)	0.843
DD (ng/ml)	1.000(0.999–1.001)	0.968	1.016(1.010–1.023)	<0.001	1.017(1.011–1.023)	<0.001
FIB (mg/dL)	0.996(0.986–1.006)	0.434	0.999(0.991–1.006)	0.753	1.000(0.994–1.006)	0.960
GLU (mmol/L)	1.059(0.760–1.475)	0.736	0.966(0.714–1.309)	0.825	1.023(0.795–1.315)	0.862
BUN (mmol/L)	1.101(0.791–1.533)	0.569	1.136(0.902–1.430)	0.277	1.066(0.909–1.251)	0.431
UA (μmol/L)	1.005(0.998–1.012)	0.177	1.007(1.000–1.014)	0.036	1.002(0.996–1.008)	0.497
TG (mmol/L)	0.428(0.210–0.871)	0.019	0.326(0.179–0.594)	<0.001	0.732(0.510–1.049)	0.089
LDL (mmol/L)	1.001(0.560–1.787)	0.998	1.064(0.536–2.112)	0.859	1.140(0.619–2.097)	0.675

Abbreviations: sIAD = spontaneous intracranial artery dissection; IS = ischemic stroke; ICH = intracerebral hemorrhage; OR = odds ratio; 95%CI = 95% confidence interval; SBP = systolic blood pressure; DBP = diastolic blood pressure; WBC = white blood cell count; NEUT = neutrophil count; PT = prothrombin time; TT = thrombin time; ALT = alanine aminotransferase; AST = aspartate aminotransferase; DD = D-dimer; FIB = fibrinogen; GLU = glucose; BUN = blood urea nitrogen; UA = uric acid; TG = triglyceride; LDL = low-density lipoprotein (please refer to Supplementary Table 2 for complete data due to space limitations).

### 3.1. Patients with sIAD versus non-IAD IS patients

**Figure 3.**
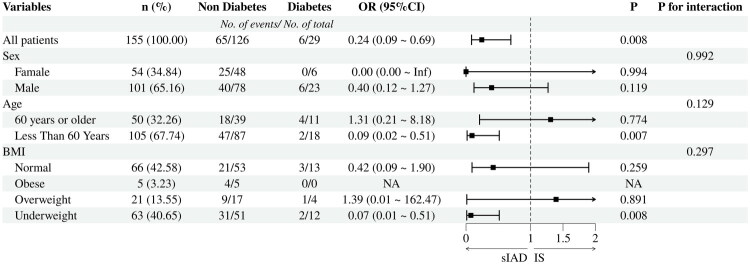
Subgroup analysis of the association between diabetes and sIAD grouped by age, sex, and BMI. Abbreviations: sIAD = spontaneous intracranial artery dissection; IS = ischemic stroke; OR = odds ratio; 95%CI= 95%confidence interval; BMI = body mass index (less than 18.5 kg/m^2^ was considered underweight, 18.5 kg/m^2^-24 kg/m^2^ was considered normal, 24kg/m^2^-28kg/m^2^ was considered overweight, and more than 28 kg/m^2^ was considered obese). Admission season, history of hypertension, history of coronary heart disease, and hypercholesterolemia were included in the model as covariates.

After screening and adjusting the pre-selected variables, patients with diabetes mellitus was associated with a reduced likelihood of sIAD (OR = 0.145, 95% CI =0.030–0.702) than non-IAD IS. In terms of clinical characteristics, the likelihood of sIAD is reduced as the patient’s admission heart rate increases. (OR = 0.944, 95% CI = 0.893–0.998). With the increase of triglyceride (TG) value, the likelihood of sIAD was lower than that of non-IAD IS (OR = 0.428, 95% CI = 0.210–0.871). In the subgroup analysis of age, sex and BMI values in the sIAD group and the IS group with diabetes as the exposure factor, the findings showed that there was no significant interaction (*P* for interaction > 0.05, Figure 3).

### 3.2. Patients with sIAD versus non-IAD ICH patients

Comparative analysis of IAD patients with non-IAD ICH patients showed that age, systolic blood pressure (SBP), and several serological indicators showed statistically significant differences. Among them, compared to patients with non-IAD stroke, those with IAD were significantly younger (OR = 0.911, 95% CI = 0.855–0.970), had lower SBP (OR = 0.941, 95% CI = 0.900–0.983). Prothrombin time (PT, OR = 0.197, 95% CI = 0.103–0.378) and TG (OR = 0.326, 95% CI = 0.179–0.594) showed the same trend. On the contrary, D-dimer (DD, OR = 1.016, 95% CI = 1.010–1.023) and uric acid levels (UA, OR = 1.007, 95% CI = 1.000–1.014) were modestly higher among IAD cases.

### 3.3. Patients with non-IAD IS versus non-IAD ICH patients

In the comparative analysis of patients with non-IAD IS group and the non-IAD ICH group, it can be seen that compared with the previous group, the statistically significant differences were also age (OR = 0.921, 95%CI = 0.868–0.976), SBP (OR = 0.962, 95% CI =0.930–0.995), PT (OR = 0.239, 95% CI = 0.129–0.444). In addition, there were significant differences in thrombin time (TT, OR = 1.246, 95%CI = 1.086–1.429) and DD (OR = 1.017, 95%CI = 1.011–1.023) between the two groups. With the increase of TT values and DD values, the likelihood of non-IAD IS was higher than that of non-IAD ICH.

### 3.4. Patients with intracranial internal carotid artery dissection vs patients with intracranial vertebral artery dissection

According to the location of the dissection, we divided the IAD group into the internal carotid artery dissection group and the vertebral artery dissection group (Supplement Table 1). The comparative analysis of the two groups showed that the probability of vertigo in patients with intracranial internal carotid artery dissection was 5.960 times higher than that in patients with intracranial vertebral artery dissection (OR = 5.960, 95% CI = 1.281–27.726). In the serologic data, the likelihood of internal carotid artery dissection occurring relative to vertebral artery dissection was increased with higher values of activated partial thromboplastin time (APTT, OR = 1.541, 95% CI = 1.128–2.105), and alanine aminotransferase (ALT, OR = 0.938, 95% CI = 0.881–0.999) showed the opposite trend (Supplementary Table 4) (complete data is set out in Supplementary Table 3).

## 4. Discussion

Our study compared sIAD patients, non-IAD IS patients, and non-IAD ICH patients in terms of possible risk factors and clinical features. We noted that the incidence of diabetes in the sIAD group was lower than that in the non-IAD IS group, which was similar to previous observational studies [20]. However, sIAD did not show this variability when grouped according to specific categories. Diabetes causes collagen cross-linking in blood vessel walls, leading to loss of collagen elasticity and consequently reduced arterial compliance [21,22]. Based on this, we reasonably speculate that the reduction of arterial compliance is more likely to promote the occurrence of IS than sIAD, but this speculation needs to be further confirmed by more experimental data. In addition, subgroup analyses showed that the likelihood of sIAD was lower than that of IS in the diabetic population, especially in patients younger than 60 years old and underweight. The gradual aging of the human being and the long-term shear force of blood flow cause a gradual thinning of the elastic layer within the arterial walls, which in turn eventually leads to blood invasion and the formation of hematomas [23]. It has been shown that there is a correlation between higher BMI and insufficient blood perfusion to the brain [24], which may suggest that cerebral blood flow perfusion volume affects the likelihood of sIAD and IS.

The distribution of other vascular risk factors such as hypertension, hypercholesterolemia, obesity and overweight (inferred from BMI) showed no difference among the three groups. In terms of the age of patients with onset of disease, both the sIAD group and the non-IAD IS group showed a trend toward younger age relative to the ICH group, yet the sIAD group did not show heterogeneity in ORs compared to the non-IAD IS group in terms of age, as has been found in previous large-scale studies [20,25], which may be related to the small size of our sample.

In terms of clinical characteristics, the sIAD group and the non-IAD IS group showed the same trend in the systolic blood pressure at the time of patient admission when compared with the non-IAD ICH group, that is, the incidence decreased with the increase of systolic blood pressure, which was consistent with the cognition of hypertension as the main cause of cerebral hemorrhage [17,26]. In addition, the serological data of the patient at the first admission attracted our attention. Among them, TG, which is closely related to the occurrence of atherosclerosis, showed a decrease in incidence with increasing values when the sIAD group was controlled against the IS and ICH groups, respectively. Elevated TG values increase the activity of cholesteryl ester transfer proteins (CETPs), which in turn leads to more TG being transferred from very-low-density lipoprotein (VLDL) to high density lipoprotein (HDL) and low density lipoprotein (LDL), in which it is further hydrolyzed by liver lipase and lipoprotein lipase (LPL) to form small and dense (higher density and smaller volume than normal) HDL and LDL particles [27]. Of the two, small and dense HDL is easily excreted from the kidney, while small and dense LDL is not easily metabolized by the liver [28,29], which will stay in the blood vessels for a longer time and is more likely to deposit on the blood vessel wall, leading to the occurrence of atherosclerotic lesions [30]. This explains our statistical analysis results and also provides a reference for us in the differential diagnosis of the three groups of patients.

When the sIAD group was compared with the non-IAD ICH group, the increased incidence of sIAD was also associated with relatively low PT values and relatively high values of DD, and UA. These serological data may provide a reference for us to identify the two in clinical practice. Among them, the increase of UA level has been considered as a sign of increased risk of cardiovascular disease [31,32]. Similar results have been obtained in previous studies [33–35]. Uric acid, a metabolite produced from the breakdown of DNA and RNA in the purine metabolic pathway, is accompanied by reactive oxygen species (ROS). Therefore, the increase of uric acid can lead to the enhancement of oxidative stress, which in turn induces DNA damage, cytotoxicity and apoptosis [36]. All of these occurrences in the vascular endothelium cells will lead to endothelial dysfunction, which in turn leads to the formation of interendothelial gaps, increased paracellular permeability, and the subsequent recruitment and infiltration of inflammatory cells, which in turn induces the formation of dissection [37]. The mechanism of other serological data (including values of TT and DD) affecting the occurrence of cerebral artery dissection is temporarily unknown, but we speculate that it may be related to inflammatory cell infiltration, endothelial cell dysfunction and other reasons.

For the two classifications of sIAD, intracranial artery dissection and vertebral artery dissection, it is noteworthy that vertigo showed a statistical difference in the regression model between the two groups. The probability of vertigo in patients with intracranial vertebral artery dissection is significantly higher than that in patients with intracranial internal carotid artery dissection. Predictably, no differences were demonstrated between the two groups in terms of vascular risk factors such as age and hypertension. In terms of serology, the increased incidence of intracranial vertebral artery dissection is associated with the relatively high APTT value and the relatively low ALT value, which provided a reference point for differentiating between the two until we had definitive diagnostic evidence from imaging and other sources.

For the imaging data we collected on 71 patients with sIAD, we noted that a combination of CTA and DSA was the most common, followed by a combination of all three tests. Compared with CTA and MRA, DSA is more accurate and reliable, has a higher detection rate, and provides a clearer view of the vascular status and the full picture of the dissecting aneurysm. However, there are disadvantages such as invasiveness and the possibility of missed diagnosis when patients with IAD have normal arterial diameters [38]. In our data, the detection rate of CTA on IAD can reach 82%, which is a little lower than the detection rate of MRA (85.2%), but due to the advantages of its short duration, simple operation, and relatively inexpensive price, it is more widely used in clinical applications [39]. Compared with the two, MRA is less used in the imaging diagnosis of dissection, but it has its corresponding application value in clinic because it is not easy to be affected by skull base bone, it is clearer than CTA and has no radiation hazard [8].

### 4.1. Strengths and limitations

Our study had several strengths. Firstly, we compared the three groups, the sIAD group, the non-IAD IS group, and the non-IAD ICH group, which might provide a more reliable basis for the correlation or difference between the three groups. Secondly, we compared and analyzed the serological data of IAD patients at admission for the first time, trying to find the serological differences between the three groups, aiming to provide a strong basis for the construction of differential diagnosis model. Thirdly, we excluded patients with intracranial artery entrapment caused by direct or indirect neck trauma in an attempt to more accurately analyze the risk factors associated with this disease. Finally, sIAD cases have been reported globally mostly in the Asian region, but relevant case-control studies are still lacking. With this study, we hope to seek more reliable evidence on risk factors and clinical diagnosis related to sIAD in the Asian region, in order to fill the current data gaps of this disease in the Asian region.

There are some limitations in our research. First of all, due to the low incidence of IAD, its data sources are limited. This study does not include potential risk factors such as migraine history and quinolone use history [40–42]. Prospective studies can be conducted to ensure the integrity of data collection through questionnaires and other methods. In addition, due to the limitation of single-center data and the low incidence of diseases, the sample size of this study is small, which affects the reliability of the results. In the future, multi-center joint research can be carried out to expand the range of sample sources, extend the accumulation time of cases, and increase the sample size to enhance the statistical effectiveness of the results.

## 5. Conclusion

Our study once again observed a decreasing trend in the incidence of diabetes in IAD compared with IS and ICH, which provided further data support for the existence of this phenomenon, but the potential mechanism of this phenomenon remains to be further explored. We also explored the differences of serological indicators between groups. It is worth noting that compared with non-IAD ICH patients, IAD patients had significantly lower PT and TG levels, while DD and UA levels were significantly higher. The mechanism of these serological indicators in the differential diagnosis of the three groups of patients remains to be confirmed, but our results provide a direction for future research on related mechanisms. Our imaging data showed that DSA has a reliable advantage in the detection rate of sIAD patients, but CTA and MRA also have their own value in clinical application due to their respective characteristics.

## Supplementary Material

Supplementary material 1118.docx

## Data Availability

The data that support the findings of this study are available from the corresponding author upon reasonable request.
