# Supplementary material for: Spontaneous intracranial artery dissection: risk factors, clinical features and imaging features
Source: Ann Med. 2026 Feb 24;58(1):2634573. doi: 10.1080/07853890.2026.2634573 (PMC12934335; doi:10.1080/07853890.2026.2634573)
Supplement: Supplementary material 1118.docx [file IANN_A_2634573_SM8156.docx]

## Supplementary material

## Supplementary Table 1. Demographic, clinical, and serologic profiles of the sIAD group, the non-IAD IS group, and the non-IAD ICH group.

| Variable | sIAD  (N=71) | IS  (N=84) | ICH  (N=102) | *P* |
| --- | --- | --- | --- | --- |
| Clinical Symptoms | | | | |
| Headache | 18(25.4%) | 17(20.2%) | / | 0.448 |
| Limb Numbness | 10(14.1%) | 10(11.9%) | / | 0.687 |
| Limb Twitching | 3(4.2%) | 3(3.6%) | / | 1.000 |
| Nausea | 7(9.9%) | 11(13.1%) | / | 0.531 |
| Respiratory Rate(bpm) | 19.34±1.74 | 18.83±0.61 | / | 0.563 |
| Serology Variables | | | | |
| EO (10^9^/L) | 0.12±0.02 | 0.14±0.07 | / | 0.633 |
| BASO (10^9^/L) | 0.02±0.01 | 0.03±0.01 | / | 0.545 |
| HCT (%) | 39.83±1.03 | 39.22±1.35 | / | 0.473 |
| MCV (fL) | 91.64±1.29 | 91.93±1.09 | / | 0.726 |
| MCH (pg) | 30.52±0.52 | 30.76±0.41 | / | 0.459 |
| MCHC (g/L) | 332.88±1.84 | 334.51±1.91 | / | 0.228 |
| MPV (fL) | 9.02±0.25 | 9.38±0.31 | / | 0.072 |
| INR | 1.06±0.02 | 1.11±0.03 | 2.77±2.30 | 0.203 |
| PTTA (%) | 91.94±2.37 | 90.39±3.56 | / | 0.470 |
| APTT (s) | 29.93±0.71 | 30.35±1.00 | 28.93±2.21 | 0.490 |
| TP (g/L) | 67.17±1.53 | 65.38±1.63 | / | 0.117 |
| ALB/GLB | 1.47±0.06 | 1.39±0.06 | / | 0.068 |
| GGT (U/L) | 32.43±7.25 | 52.45±20.60 | / | 0.091 |
| ALP (U/L) | 79.21±5.72 | 88.94±10.13 | / | 0.115 |
| TBA (μmol/L) | 3.90±0.65 | 6.26±3.61 | / | 0.239 |
| CYSC (mg/L) | 0.91±0.05 | 0.99±0.12 | / | 0.197 |
| C1q (mg/L) | 172.21±9.05 | 181.44±8.85 | / | 0.151 |
| K (mmol/L) | 3.84±0.09 | 3.87±0.10 | / | 0.708 |
| Cl (mmol/L) | 105.11±0.68 | 104.21±1.06 | / | 0.158 |
| Phos (mmol/L) | 1.19±0.05 | 1.13±0.06 | / | 0.104 |

Abbreviations: sIAD=spontaneous intracranial artery dissection; IS=ischemic stroke; ICH=intracerebral hemorrhage; EO=eosinophil count; BASO=basophil count; HCT=hematocrit; MCV=mean corpusular volume; MCH=mean corpusular hemoglobin; MCHC=mean corpusular hemoglobin concerntration; MPV=mean platelet volume; INR= international normalized ratio; PTTA=prothrombin time activity; APTT=activated partial thromboplastin time; TP=total protein; ALB=albumin; GGT=γ-glutamyl transpeptidase; ALP=alkaline phosphatase; TBA=4,6-Dihydroxy-2-mercaptopyrimidine; CYSC=cystatin C; C1q=complement component 1, q fragment; K=potassium; Na=sodium; Cl=chlorine; Phos=Phosphorus.

***Supplementary Table 2. Multiple logistic regression model for the sIAD, non-IAD IS, and non-IAD ICH groups.***

| Variable | sIAD vs IS  OR (95%CI) | *P* | sIAD vs ICH  OR (95%CI) | *P* | IS vs ICH  OR (95%CI) | *P* |
| --- | --- | --- | --- | --- | --- | --- |
| Heart Rate(bpm) | 0.944(0.893-0.998) | 0.044 | 0.974(0.915-1.038) | 0.423 | 1.004(0.954-1.057) | 0.881 |
| Dizziness | 0.647(0.188-2.224) | 0.489 | / | / | / | / |
| Limb Weakness | 0.821(0.180-3.741) | 0.799 | / | / | / | / |
| Dyskinesia | 0.647(0.076-5.524) | 0.691 | / | / | / | / |
| Slurred Speech | 0.328(0.052-2.070) | 0.236 | / | / | / | / |
| Vomiting | 0.840(0.126-5.582) | 0.857 | / | / | / | / |
| RDW (%) | 0.907(0.485-1.695) | 0.759 | / | / | / | / |
| MPV (fL) | 0.699(0.430-1.138) | 0.150 | / | / | / | / |
| GGT (U/L) | 0.990(0.973-1.008) | 0.279 | / | / | / | / |
| SOD (kU/L) | 1.021(0.996-1.047) | 0.100 | / | / | / | / |
| CO_2_ (mmol/L) | 1.049(0.887-1.241) | 0.578 | / | / | / | / |
| SA (mg/L) | 1.006(0.999-1.012) | 0.098 | / | / | / | / |
| Na (mmol/L) | 1.163(0.978-1.382) | 0.088 | / | / | / | / |

Abbreviations: sIAD=spontaneous intracranial artery dissection; IS=ischemic stroke; ICH=intracerebral hemorrhage; OR=odds ratio; 95%CI=95% confidence interval; RDW=red blood cell volume distribution width; MPV=mean platelet volume; GGT=γ-glutamyl transpeptidase; SOD=superoxide dismutase; CO_2_=carbon dioxide; SA=sialic acid; Na=sodium.

## Supplementary Table 3. Demographic characteristics, clinical characteristics and serological characteristics of specific sIAD classifications.

| Variable | Intracranial vertebral artery dissection (N=34) | Intracranial internal carotid artery dissection (N=37) | *P* |
| --- | --- | --- | --- |
| Age (yr) | 53.85±4.64 | 57.00±3.36 | 0.269 |
| Sex, males | 23(67.6%) | 23(62.2%) | 0.629 |
| BMI (kg/m^2^) | 24.45±1.02 | 24.68±1.14 | 0.764 |
| Admission Season |  |  | 0.345 |
| Spring | 16(47.1%) | 10(27.0%) |  |
| Summer | 7(20.6%) | 9(24.3%) |  |
| Autumn | 6(17.6%) | 11(29.7%) |  |
| Winter | 5(14.7%) | 7(18.9%) |  |
| Hypertension | 16(47.1%) | 22(59.5%) | 0.295 |
| Diabetes | 6(17.6%) | 0 | 0.025 |
| Coronary Heart Disease | 5(14.7%) | 1(2.7%) | 0.165 |
| Hypercholesterolemia | / | / |  |
| Operation History | 6(20%) | 7(21.2%) | 0.557 |
| Heart Rate(bpm) | 75.91±3.42 | 78.54±4.40 | 0.347 |
| Respiratory Rate(bpm) | 18.62±0.73 | 20.00±3.34 | 0.434 |
| SBP (mmHg) | 131.32±6.35 | 129.19±5.62 | 0.610 |
| DBP (mmHg) | 82.06±4.73 | 82.30±4.47 | 0.941 |
| Headache | 11(32.4%) | 7(18.9%) | 0.194 |
| Dizziness | 12(35.3%) | 22(59.5%) | 0.042 |
| Disturbance Of Consciousness | / | / | / |
| Limb Numbness | 7(20.6%) | 3(8.1%) | 0.131 |
| Limb Weakness | 5(14.7%) | 7(18.9%) | 0.636 |
| Dyskinesia | 3(8.8%) | 1(2.7%) | 0.264 |
| Limb Twitching | 0 | 3(8.1%) | 0.269 |
| Slurred Speech | 1(2.9%) | 4(10.8%) | 0.406 |
| Nausea | 5(14.7%) | 2(5.4%) | 0.360 |
| Vomiting | 5(14.7%) | 2(5.4%) | 0.360 |
| Operative Treatment | 26(76.5%) | 28(75.7%) | 0.938 |
| Medication | 7(20.6%) | 8(21.6%) | 0.915 |
| Use of anticoagulant or antiplatelet drugs | 27(79.4%) | 26(70.3%) | 0.376 |
| Serology | | | |
| WBC (10^9^/L) | 5.62±0.46 | 5.69±0.48 | 0.828 |
| RBC (10^12^/L) | 4.41±0.18 | 4.31±0.17 | 0.408 |
| HGB (g/L) | 134.25±5.14 | 131.33±5.46 | 0.433 |
| PLT (10^9^/L) | 214.69±24.23 | 202.78±19.32 | 0.434 |
| NEUT (10^9^/L) | 3.54±0.40 | 3.43±0.41 | 0.714 |
| LYMPH (10^9^/L) | 1.49±0.17 | 1.64±0.16 | 0.177 |
| MONO (10^9^/L) | 0.45±0.04 | 0.47±0.04 | 0.515 |
| EO (10^9^/L) | 0.13±0.03 | 0.12±0.03 | 0.942 |
| BASO (10^9^/L) | 0.02±0.01 | 0.02±0.01 | 0.747 |
| HCT (%) | 40.41±1.53 | 39.29±1.44 | 0.283 |
| MCV (fL) | 91.76±1.37 | 91.53±2.19 | 0.862 |
| MCH (pg) | 30.50±0.57 | 30.54±0.87 | 0.935 |
| MCHC (g/L) | 332.28±2.16 | 333.43±3.02 | 0.539 |
| RDW (%) | 13.40±0.29 | 13.29±0.35 | 0.625 |
| MPV (fL) | 8.94±0.30 | 9.09±0.39 | 0.548 |
| PT (s) | 11.43±0.21 | 11.71±0.34 | 0.172 |
| INR | 1.05±0.02 | 1.07±0.03 | 0.167 |
| PTTA (%) | 93.17±2.55 | 90.81±4.00 | 0.315 |
| APTT (s) | 28.98±0.83 | 30.81±1.09 | 0.009 |
| TT (s) | 14.34±0.55 | 14.60±0.56 | 0.504 |
| FIB (mg/dL) | 297.82±24.70 | 278.73±23.27 | 0.257 |
| DD (ng/ml) | 498.29±243.21 | 247.39±118.45 | 0.065 |
| ALT (U/L) | 32.31±8.02 | 19.38±3.11 | 0.004 |
| AST (U/L) | 27.11±6.06 | 20.92±4.18 | 0.087 |
| AST/ALT | 1.06±0.21 | 1.21±0.17 | 0.281 |
| TBIL (μmol/L) | 12.35±1.94 | 12.17±1.54 | 0.883 |
| DBIL (μmol/L) | 2.61±0.34 | 2.74±0.42 | 0.649 |
| UBIL (μmol/L) | 9.73±1.76 | 9.43±1.26 | 0.775 |
| TP (g/L) | 69.20±2.40 | 65.31±1.83 | 0.011 |
| ALB (g/L) | 40.50±1.57 | 38.85±1.04 | 0.080 |
| GLB (g/L) | 28.70±1.46 | 26.46±1.41 | 0.029 |
| A/G | 1.44±0.09 | 1.50±0.08 | 0.289 |
| GGT (U/L) | 33.48±9.24 | 31.46±11.46 | 0.783 |
| ALP (U/L) | 85.07±9.37 | 73.84±6.78 | 0.053 |
| TBA (μmol/L) | 3.94±0.84 | 3.85±1.03 | 0.895 |
| SOD (kU/L) | 180.68±8.90 | 171.58±6.91 | 0.103 |
| GLU (mmol/L) | 5.61±0.44 | 5.36±0.73 | 0.572 |
| BUN (mmol/L) | 5.59±0.64 | 5.31±0.44 | 0.464 |
| CREA (μmol/L) | 69.25±6.39 | 69.38±4.92 | 0.974 |
| UA (μmol/L) | 360.62±31.63 | 341.93±24.18 | 0.339 |
| CO_2_ (mmol/L) | 26.74±1.25 | 26.23±1.16 | 0.545 |
| CYSC (mg/L) | 0.90±0.07 | 0.91±0.07 | 0.897 |
| SA (mg/L) | 590.31±27.90 | 564.75±38.96 | 0.290 |
| C1q (mg/L) | 179.88±15.34 | 165.16±10.36 | 0.106 |
| CHOL (mmol/L) | 3.76±0.47 | 3.84±0.43 | 0.787 |
| TG (mmol/L) | 1.33±0.25 | 1.34±0.22 | 0.927 |
| HDL (mmol/L) | 1.06±0.08 | 1.09±0.07 | 0.587 |
| LDL (mmol/L) | 2.17±0.35 | 2.16±0.31 | 0.991 |
| K (mmol/L) | 3.83±0.14 | 3.85±0.12 | 0.764 |
| Na (mmol/L) | 140.29±0.86 | 141.16±0.78 | 0.127 |
| Cl (mmol/L) | 104.38±0.88 | 105.78±1.02 | 0.039 |
| Ca (mmol/L) | 2.29±0.03 | 2.26±0.03 | 0.195 |
| Mg (mmol/L) | 0.90±0.03 | 0.90±0.03 | 0.800 |
| Phos (mmol/L) | 1.17±0.06 | 1.21±0.07 | 0.438 |

Note: Abbreviations: sIAD= spontaneous intracranial artery dissection; SBP=systolic blood pressure; DBP=diastolic blood pressure; WBC=white blood cell count; RBC=red blood cell; HGB=hemoglobin; PLT=platelet count; NEUT=neutrophil count; LYMPH=lymphocyte count; MONO=monocyte count; EO=eosinophil count; BASO=basophil count; HCT=hematocrit; MCV=mean corpusular volume; MCH=mean corpusular hemoglobin; MCHC=mean corpusular hemoglobin concerntration; RDW=red blood cell volume distribution width; MPV=mean platelet volume; PT=prothrombin time; INR= international normalized ratio; PTTA=prothrombin time activity; APTT=activated partial thromboplastin time; TT=thrombin time; FIB= fibrinogen; DD=D-dimer; ALT=alanine aminotransferase; AST=aspartate aminotransferase; TBIL=total bilirubin; DBIL=direct bilirubin; UBIL=urinary bilirubin; TP=total protein; ALB=albumin; GLB=globulin; GGT=γ-glutamyl transpeptidase; ALP=alkaline phosphatase; TBA= 4,6-Dihydroxy-2-mercaptopyrimidine; SOD=superoxide dismutase; GLU=glucose; BUN=blood urea nitrogen; CREA=creatinine; UA=uric acid; CO_2_=carbon dioxide; CYSC=cystatin C; SA=sialic acid; C1q=complement component 1, q fragment; CHOL=cholesterol; TG=triglyceride; HDL=high-density lipoprotein; LDL=low-density lipoprotein; K=potassium; Na=sodium; Cl=chlorine; Ca=Calcium; Mg=Magnesium; Phos=Phosphorus.

## Supplementary Table 4. Correlation analysis between variables and specific subgroups of sIAD patients.

| Variable | Intracranial vertebral artery dissection VS Intracranial internal carotid artery dissection  OR (95%CI) | *P* |
| --- | --- | --- |
| Vertigo | 5.960(1.281-27.726) | 0.023 |
| Diabetes | 0 | 0.999 |
| APTT (s) | 1.541(1.128-2.105) | 0.007 |
| DD (ng/ml) | 0.998(0.996-1.000) | 0.120 |
| ALT (U/L) | 0.938(0.881-0.999) | 0.045 |
| AST (U/L) | 1.007(0.945-1.074) | 0.823 |
| TP (g/L) | 0.995(0.824-1.201) | 0.960 |
| ALB (g/L) | 0.785(0.550-1.120) | 0.181 |
| ALP (U/L) | 1.007(0.964-1.051) | 0.763 |
| Cl (mmol/L) | 0.992(0.739-1.331) | 0.955 |

Abbreviations: sIAD=spontaneous intracranial artery dissection; OR=odds ratio; 95%CI=95%confidence interval; APTT=activated partial thromboplastin time; DD=D-dimer; ALT=alanine aminotransferase; AST=aspartate aminotransferase; TP=total protein; ALB=albumin; ALP=alkaline phosphatase; Cl=chlorine.
